# Evaluation of accelerated real-time CMR using sparse sampling with iterative SENSE reconstruction in patients and volunteers

**DOI:** 10.1186/1532-429X-16-S1-O12

**Published:** 2014-01-16

**Authors:** Bradley D Allen, Maria Carr, Michael O Zenge, Michaela Schmidt, Mariappan S Nadar, Bruce S Spottiswoode, Jeremy D Collins, James C Carr

**Affiliations:** 1 Radiology, Northwestern University, Chicago, Illinois, USA; 2 Siemens AG Healthcare Sector, Erlangen, Germany; 3 Siemens Corporate Technology, Princeton, New Jersey, USA; 4 Siemens Healthcare USA, Inc., Chicago, Illinois, USA

## Background

The use of gated CMR can be limited by motion artifacts secondary to cardiac and respiratory motion. Imaging is especially challenging in patients with arrhythmias or those who cannot perform adequate breath-holds. Real-time CMR is a non-gated technique that has been successfully applied in scenarios where standard segmented acquisitions break down. In this study, we sought to accelerate real-time acquisition by using sparse sampling with an iterative SENSE reconstruction.

## Methods

**Figure 1:**
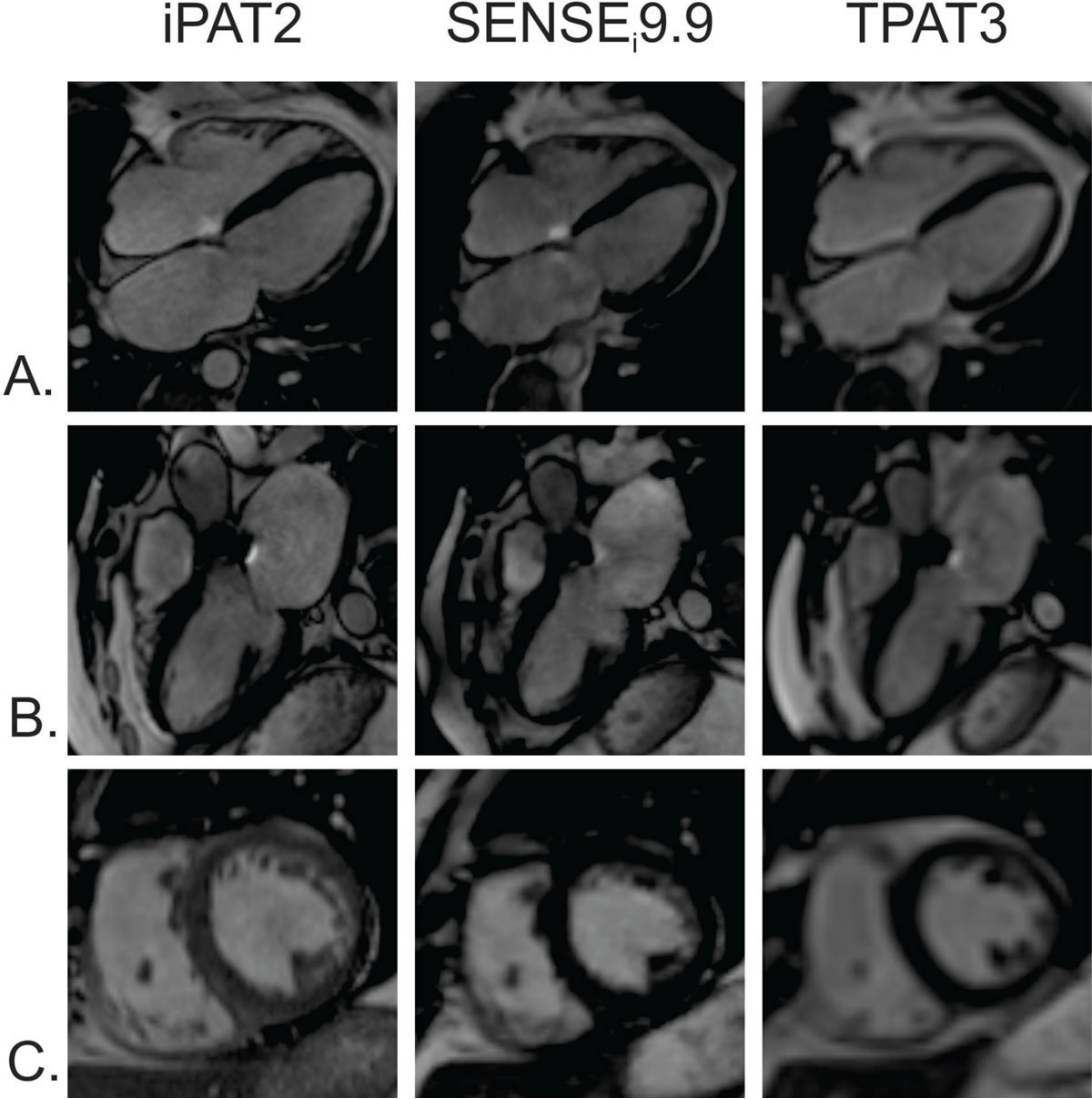
**Visual comparison of the standard segmented (iPAT2), accelerated real-time iterative SENSE reconstruction (SENSE_i_9.9), and accelerated real-time (TPAT3) CMR in a 73 year-old patient undergoing imaging for post-operative aortic valve replacement**. 4-chamber (A), 3-chamber (B), and mid-short axis slices (C) are shown.

Seven consecutively recruited patients undergoing non-emergent CMR (58 ± 18 years, M:F = 3:4) and 6 volunteers (38 ± 11 years, M:F = 4:2) were included in this IRB-approved study. CMR was performed at 1.5T (MAGNETOM Aera, Siemens Healthcare, Erlangen, Germany). The examination included acquisitions of standard segmented SSFP (iPAT2) (GRAPPA accel factor 2, TR 40 msec, 2.1 × 2.1 × 10 mm^3^) cine, standard real time (TPAT3) (TPAT accel factor 3, TR 62 msec, 2.9 × 2.9 × 7 mm^3^), and the investigational prototype sparsely sampled SSFP with iterative SENSE reconstruction with L1 regularization along one spatial and temporal dimension (SPARSE_i_9.9) (accel factor 9.9, TR 43 msec, 2.0 × 2.0 × 7 mm^3^) (1). Each technique was used to acquire a three-, four-chamber, and short axis series in identical slice positions (Figure 1), with coverage of the entire left ventricle (LV) and 10 mm interslice gaps. Individual slice scan times were recorded. Quantitative LV functional analysis was performed. A reviewer blinded to acquisition type scored images for overall image quality, noise, and artifacts using a 5-point Likert scale. Continuous variables were compared between groups using a paired t-test, and ordinal variables were compared using a Wilcoxon signed-rank test.

## Results

**Figure 2:**
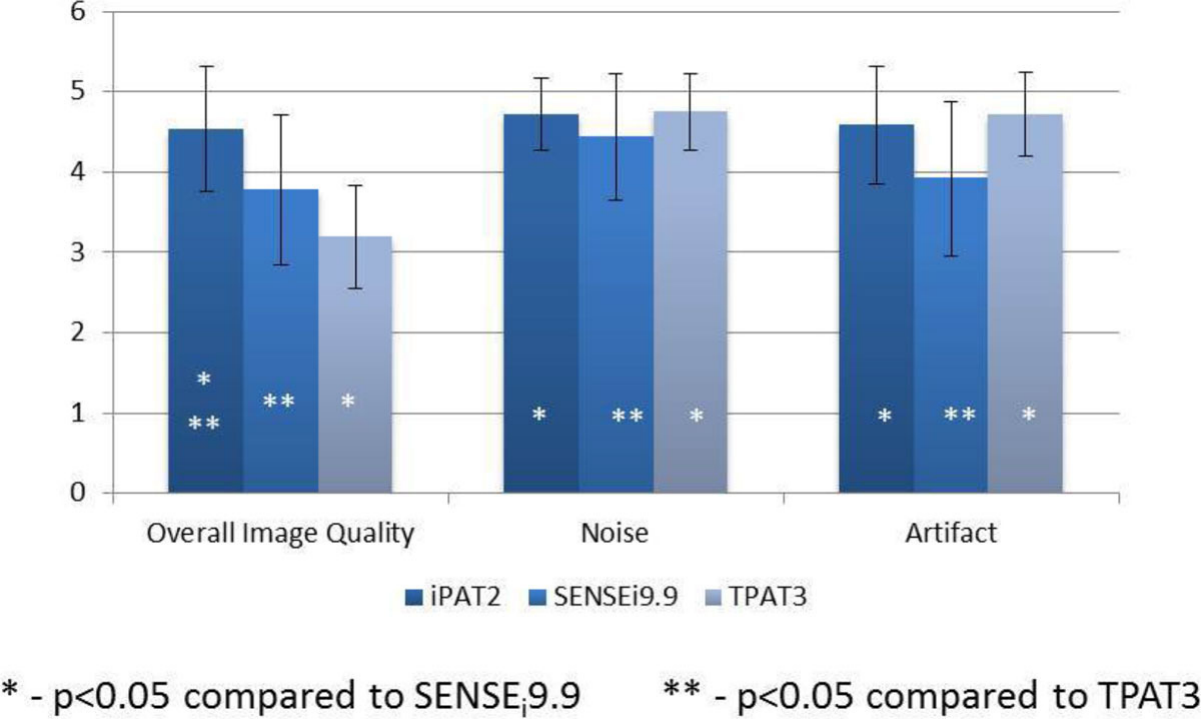
**Qualitative analysis in patients and volunteers**.

In a combined analysis of patients and volunteers, there was no significant difference between LV ejection fraction between iPAT2 and SPARSE_i_9.9 (p = 0.18) or TPAT3 (p = 0.31), and there was no difference between either real time acquisition (p = 0.83). The iPAT2 technique measured higher myocardial mass than SPARSE_i_9.9 (105 ± 25 g vs. 95 ± 30 g, p = 0.004) and TPAT3 (86 ± 26 g, p < 0.001). The iPAT2 technique was superior to both SPARSE_i_9.9 (p < 0.001) and TPAT3 (p < 0.001) in overall image quality. The SPARSE_i_9.9 group had higher image quality compared to TPAT3 (p < 0.001), but TPAT3 had marginally reduced noise (p = 0.01) and reduced artifact (p < 0.001). (Figure 2) Short axis slice acquisition times were shorter for SPARSE_i_9.9 (3.8 ± 0.6 sec) than iPAT2 (8.9 ± 1.5 sec, p < 0.001) and TPAT3 (5.7 ± 1.0 sec, p < 0.001).

## Conclusions

Highly accelerated real-time CMR using sparse sampling with iterative SENSE reconstruction can be successfully applied in patients and volunteers with accurate calculation of LV functional parameters. Image quality is reduced relative to gold-standard segmented acquisitions, but is superior to standard real-time acquisitions. 1. Liu J, et al. ISMRM 20th Annual Meeting. Melbourne, Australia, 2012:4249.

## Funding

NIH NCI 5R25CA132822-04.

